# An Unusual Presentation of Herpes Esophagitis in an Immunocompromised Individual

**DOI:** 10.7759/cureus.15635

**Published:** 2021-06-14

**Authors:** Riya Kataria, Lawrence D'Cruze, Tusharindra Lal, N. Senthil, Sandhya Sundaram

**Affiliations:** 1 Medicine, Sri Ramachandra Institute of Higher Education and Research, Chennai, IND; 2 Pathology, Sri Ramachandra Institute of Higher Education and Research, Chennai, IND; 3 Internal Medicine, Sri Ramachandra Institute of Higher Education and Research, Chennai, IND

**Keywords:** herpetic esophagitis, opportunistic infections, infectious esophagitis, viral infection, herpes pathology

## Abstract

Herpes simplex infection remains the third most common cause of esophagitis following gastric reflux disease and candida infection. This disease usually occurs in immunocompromised individuals; however, it has been frequently reported in healthy individuals. We present a case of a 39-year-old man who presented to the ER with symptoms unusual of herpes esophagitis. He was presumed to be immunocompromised due to uncontrolled diabetes mellitus and chronic alcohol use. Endoscopy revealed features in favor of candidiasis; however, histopathology displayed characteristic features of herpes infection. Herpes esophagitis should thus be suspected in immunocompromised patients with an independent underlying pathology and treated early with antiviral agents like acyclovir to prevent impending complications.

## Introduction

Herpes simplex virus (HSV) has been recognized as an opportunistic invader of the esophagus in immunosuppressed, immunocompromised, or severely ill subjects [1]. It has been reported that the presentation of herpes simplex esophagitis (HSE) can often overlap with symptoms of cytomegalovirus (CMV) esophagitis [2], esophageal candidiasis [3], and gastric reflux, posing a diagnostic challenge.

We describe a case of a 39-year-old male with uncontrolled diabetes and a history of alcohol consumption, who presented with symptoms inconsistent with HSE. We believe that a mere absence of conventional signs and symptoms does not exclude the diagnosis of HSE and it requires a high index of suspicion, especially while treating immunocompromised individuals.

## Case presentation

A 39-year-old man presented with complaints of upper abdominal pain, nausea, and multiple episodes of vomiting for the past seven days, suggestive of gastritis. There was no history of dysphagia, odynophagia, and chest pain. The patient was a known case of diabetes mellitus with a history of alcohol consumption for the past 10 years. There were no risk factors suggestive for HIV and a negative history of any recent corticosteroid use.

Vital signs were unremarkable. On examination, abdominal guarding was reported in the left hypochondrium. Complete blood count (CBC) revealed normal hemoglobin levels, leukocytopenia (WBC = 2,400/mm^3^) with elevated neutrophils, decreased lymphocytes, thrombocytopenia (100,000/mm^3^), increased serum amylase (358U/L), serum lipase (957U/L), and elevated creatinine levels (3.4mg/dL). HbA1c levels were also raised (10.2%), indicating poor diabetic control.

On ultrasonography (USG) of the abdomen, an increased pancreatic volume with a marked decrease in echogenicity was reported, while the computed tomography (CT) scan revealed the presence of peripancreatic fluid, both typical of acute pancreatitis. However, considering the history of chronic alcoholism and on further evaluation, an acute exacerbation of chronic pancreatitis was established. An upper gastrointestinal endoscopy was performed, which revealed longitudinal, nodular, white plaques with ulceration, and raised margins in the middle and lower thirds of the esophagus. Multiple biopsies were taken from various sections of the esophagus and sent for histopathology. This appearance was indicative of esophageal candidiasis; however, no fungal organisms were identified.

**Figure 1:**
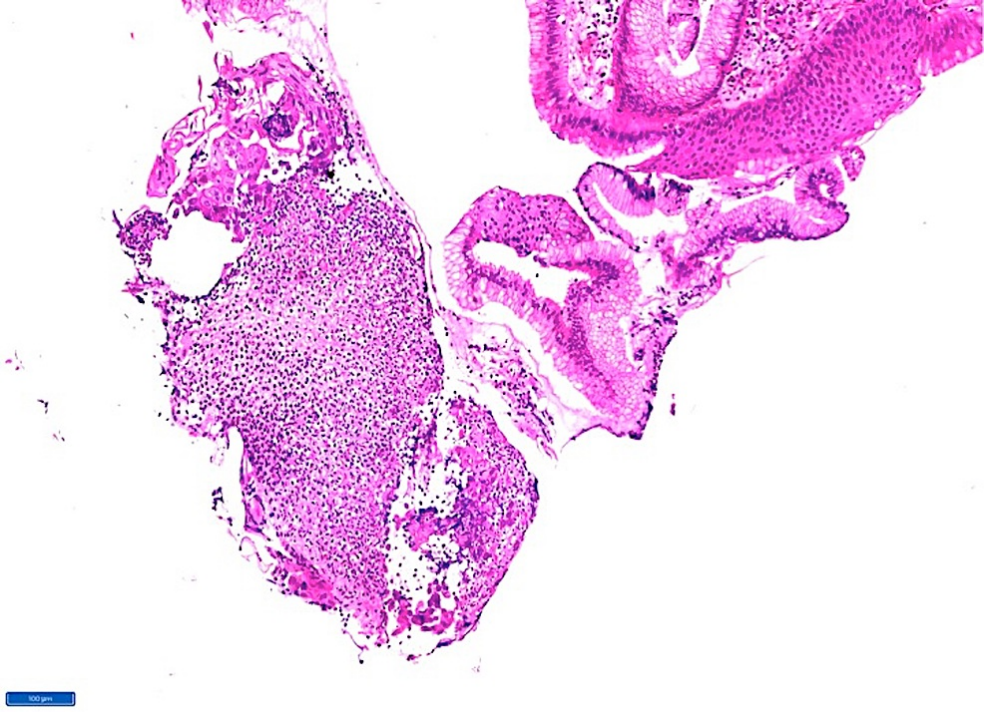
Microphotograph (hematoxylin and eosin stain x100) showing gastro-esophageal junctional tissue with dense inflammatory infiltrate composed predominantly of polymorphs

Histopathological examination (HPE) of the ulcerative focus displayed an exudative and inflammatory infiltrate composed predominantly of neutrophils (Figure 1).

**Figure 2:**
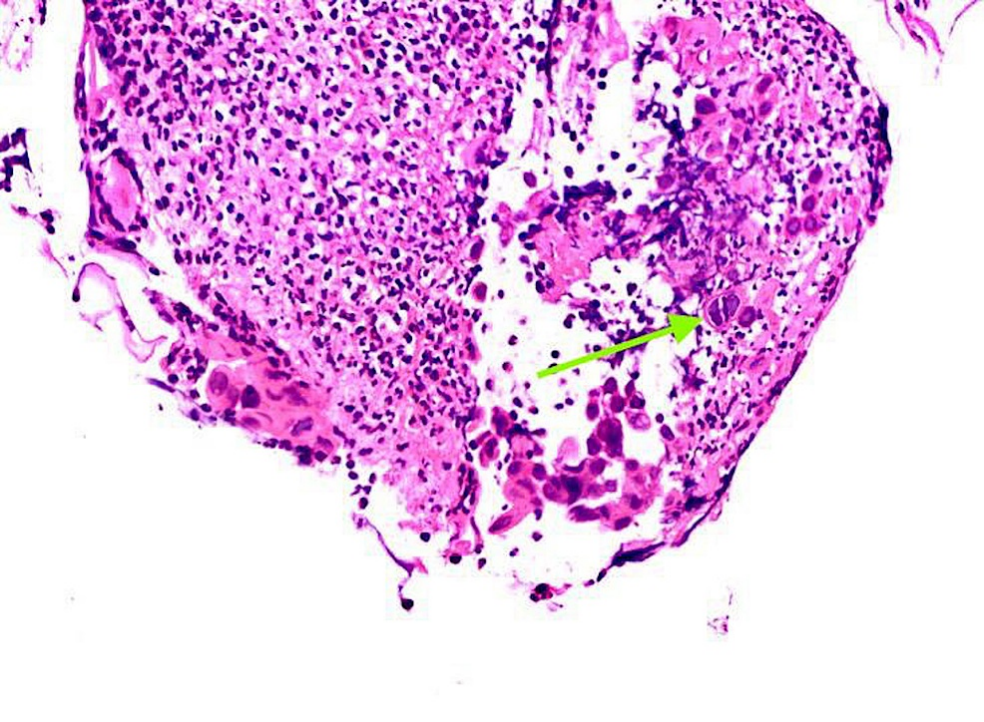
Higher magnification (H&E staining x200) showing HSV inclusions (arrow) with characteristic multinucleation, margination, and molding H&E - hematoxylin and eosin, HSV - herpes simplex virus

The gastro-esophageal junction revealed multinucleated giant cells with nuclear molding and “ground glass” appearance of the chromatin features characteristic of herpes simplex infection (Figure 2).

After diagnostic confirmation, the patient was started on intravenous acyclovir for two weeks and subjected to other symptomatic treatment. He was started on pancreatic enzyme replacement therapy and insulin. Creatinine levels returned to normal and he was then started on oral antidiabetics (sulfonylureas and metformin). At the time of discharge, labs were repeated and liver function test (LFT), aminotransferase (AST), and alanine transaminase (ALT) levels had returned to normal values. Following a psychiatric consult, benzodiazepines were given for alcohol de-addiction and he was suggested to undergo rehabilitation therapy. The patient had an uneventful recovery and was discharged after the resolution of symptoms.

## Discussion

Infectious esophagitis is the third leading cause of esophagitis, followed by gastro-esophageal reflux disease and eosinophilic esophagitis [4]. After Candida albicans (93%), HSV type-1 (0.5%-6%) remains the most common etiology to involve the visceral organs of an immunocompromised patient [3,5]. There are several known risk factors for the development of HSE, including hematologic malignancy, solid tumors, extensive burns, autoimmune disease, and HIV infection [6]. In our case, the patient was a known diabetic (non-compliant) and elevated blood sugars tend to have a negative effect on the immune system making the individual immunocompromised [7]. However, with recent advances in diagnostic procedures, many well-documented cases of HSE have been reported in healthy individuals as well [8-11].

HSV infection of visceral organs can occur due to viremia, leading to the involvement of multiple organs. In most cases, it occurs as a result of reactivation of underlying infection and further spreads to the esophagus through the vagus nerve or by direct extension from the oral cavity [12]. Clinically, HSE typically presents with odynophagia, dysphagia, fever, severe chest or retrosternal pain and gastrointestinal bleeding [13] with preceding or coexistent herpes labialis, or oropharyngeal ulcers. In contrast to the classical symptoms, the atypical presentation, as seen in our case is seldom reported in the existing literature.

Endoscopy remains the investigation of choice, which is further complemented by HPE and viral culture. In the majority of the cases (>50%), the lesions involve the distal or mid-esophagus while the remaining may affect the entire esophagus [14]. In the early stages, vesicles may be seen, which further slough off to form discrete, circumscribed ulcers with raised edges and friable changes in the mucosa [1]. However, the index case showed deviation from these standard findings.

A presumptive diagnosis of HSE based on endoscopic findings alone can be challenging. Differentiation between CMV and herpes simplex is essentially important since potential treatment with acyclovir has shown to be effective for HSV alone [15].

In some cases where HSE is suspected, a biopsy should be obtained from the edges of the ulcer and sent for HPE as well as viral culture. Virus isolation by cell culture has traditionally been considered the diagnostic “gold standard” for HSV infection [1]. The characteristic histologic appearance of multinucleated giant cells with eosinophilic intranuclear inclusions called Cowdry type A inclusions and nuclear chromatin with a ground-glass appearance is suggestive of HSV infection [16,17]. In recent years, HSV DNA-PCR is considered the most sensitive, cost-effective, rapid, and easiest diagnostic tool of HSV infection [18].

Wang et al. reported that though the clinical symptoms are similar, immunocompetent individuals are diagnosed at a younger age and have a faster recovery with minimal complications, as opposed to immunocompromised individuals [8].

In addition to the unusual endoscopic findings, our patient had an atypical clinical presentation, which makes this case unique and noteworthy. The diagnosis of herpes esophagitis is made predominantly with the help of endoscopy; however, histopathology, viral cultures, and PCR are crucial to establish a definitive diagnosis. The use of antiviral agents like acyclovir, famciclovir, and valacyclovir is important in the initial stages of the disease to hasten recovery and rapidly achieve symptomatic relief [19]. An early diagnosis and treatment with antiviral agents are imperative to prevent major complications such as spontaneous esophagal perforation, mediastinitis, and gastrointestinal bleeding [7].

## Conclusions

The presentation of herpes infection is wide and varied. The discordance between clinical presentation and investigative evidence of herpes esophagitis can interfere with an accurate diagnosis. An endoscopic investigation followed by confirmation on histopathology helps in rendering the diagnosis. However, viral cell culture and DNA-PCR may be additionally performed, especially in cases presenting with atypical signs and symptoms. The current case is documented for its rare presentation. Early diagnosis and treatment with acyclovir help in rapid recovery and in preventing various complications.
